# Supplementary material for: Usage of Digital Health Tools and Perception of mHealth Intervention for Physical Activity and Sleep in Black Women
Source: Int J Environ Res Public Health. 2022 Jan 29;19(3):1557. doi: 10.3390/ijerph19031557 (PMC8834665; doi:10.3390/ijerph19031557)
Supplement: Supplementary file 1 [file ijerph-19-01557-s001.zip › ijerph-1532503-supplementary.pdf]

UNIVERSITY OF TEXAS ARLINGTON

Physical Activity and Sleep in Reproductive-Age Black Women

Thank you for taking the time to complete this survey.

How do you feel about your current physical activity level?

- ◯ Happy with it - I am doing what I think I need to do
- ◯ I need to be more active
- ◯ I am trying to cut back on my activity level
- ◯ Other (please explain)

Think about your physical activity level before the COVID-19 pandemic and compare it with your current physical activity level:

- ◯ I am much less active right now than pre-COVID
- ◯ I am a little bit less active right now than pre-COVID
- ◯ My activity level is about the same
- ◯ I am a little bit more active right now than pre-COVID
- ◯ I am much more active right now than pre-COVID

Do you exercise three times a week for at least 20 minutes each time?

- ◯ Yes, I have been for more than 6 months
- ◯ Yes, I have been for less than 6 months
- ◯ No, but I am planning to start in the next 30 days
- ◯ No, but I am planning to start in the next 6 months
- ◯ No, and I don't plan to start in the next 6 months

Moderate-intensity activities are those that get you moving fast enough or strenuously enough to burn off three to six times as much energy per minute as you do when you are sitting quietly. Some examples of moderate-intensity exercise include fast walking, easy bicycling, hiking uphill, gardening and some housework such as vacuuming.

Considering the definition above, during the past 30 days, how many minutes of moderate-intensity activities you typically do in a week?

- 0-15 minutes / week
- 15-30 minutes / week
- 30-90 minutes / week
- 90-150 minutes / week
- 150-200 minutes / week
- More than 200 minutes

Would you be interested in an in-person program (that is, you will need to go visit a place in-person from time to time) that helps you to become and stay more active in your daily life?

- Yes
- Yes, but only when the COVID-19 pandemic is over
- No
- Not sure

Please explain

Would you be interested in a remotely delivered program (such as through the use of website, emails, text messages, smartphone apps) that helps you to become and stay more active in your daily life?

- ◯ Yes
- ◯ No
- ◯ Not sure

Please explain:

During the past month, how many hours of actual sleep did you get at night? (This might be different than the number of hours you spend in bed.)

Please put in the typical hours per night:

During the past month, how would you rate your sleep quality overall?

- ◯ Very good
- ◯ Fairly good
- ◯ Fairly bad
- ◯ Very bad

During the past month, how much of a problem has it been for you to keep up enough enthusiasm to get things done?

- ◯ No problem at all
- ◯ Only a very slight problem
- ◯ Somewhat of a problem
- ◯ A very big problem

During the past month, how often have you taken medicine to help you sleep (prescribed or "over the counter")?

- ◯ Not during the past month
- ◯ Less than once a week
- ◯ Once or twice a week
- ◯ Three or more times a week

Would you be interested in non-pharmaceutical approaches (meaning *NOT* to take any medicines) to improve your sleep?

- ◯ Yes
- ◯ No
- ◯ Not sure
- ◯ I don't need it. I don't have any problems with my sleep.

Consider your overall sleep before the start of the COVID-19 pandemic and compare it with your current sleep:

- [ ] I sleep more than pre-COVID
- [ ] I get about the same amount of sleep
- [ ] I sleep less than pre-COVID
- [ ] I sleep worse than pre-COVID
- [ ] My sleep quality is about the same
- [ ] I sleep better than pre-COVID

Have you ever owned a wearable fitness tracker or smartwatch such as Fitbit, Apple Watch, Garmin?

- ○ Yes
- ○ No, but planning to get one in the next few months
- ○ No, and not planning to get one in the next few months

Please list all the fitness tracker or smartwatch you have owned:

How long have you been using each of the Fitness tracker or smartwatch?

What are your primary goals for using the fitness tracker or smartwatch? [check all that apply]

- ☐ To track my activity levels
- ☐ To tell time
- ☐ To receive notifications from my phone (e.g., text messages, phone calls, calendar, etc.)
- ☐ Just for fun
- ☐ Other

Which fitness tracker or smartwatch are you planning to get?

What will be your primary goals for using the fitness tracker or smartwatch? [check all that apply]

- ☐ To track my activity levels
- ☐ To tell time
- ☐ To receive notifications from my phone (e.g., text messages, phone calls, calendar, etc.)
- ☐ Just for fun
- ☐ Other

Do you own a smartphone?

- ○ Yes, I have an iPhone
- ○ Yes, I have an Android phone
- ○ No

During the past 12 months, how often did you use health-related apps (e.g., Apple's Health, Google Fit, MyFitnessPal) on your smartphone?

- Almost daily
- Couple times a week
- Couple times a month
- Couple times a year
- Never

Please list the health-related apps that you are currently using on your smartphone:

During the past 12 months, have you ever used your smartphone to look up information about a health condition?

- Yes
- No

Would you be interested in receiving personalized feedback messages based on a wearable device about your activity levels?

- Yes
- No
- Not sure

Would you be interested in receiving personalized feedback messages based on a wearable device about your sleep?

- ◯ Yes
- ◯ No
- ◯ Not sure

Would you ever be willing to give your blood samples for health research purposes?

- ◯ Yes
- ◯ No
- ◯ Not sure

Please explain:

Please indicate which of the following concerns you have about providing blood samples for research studies: (check all that apply)

- ☐ I don’t trust the health researchers with my blood samples
- ☐ I fear that my samples may be misused or used for profit
- ☐ I fear that health researchers will provide my samples to the government
- ☐ It is too much information for health researchers to know about me
- ☐ Other concerns (please specify)

What would increase the likelihood of you giving blood samples for research purposes? (check all that apply)

- ☐ The research team is clear what the blood samples are being used for
- ☐ The research team shares my blood sample results with me
- ☐ When I know my samples would help to advance health research
- ☐ The research team pays me for my time and effort
- ☐ The research team members include people who look like me
- ☐ Other (please specify)

What is your sex at birth?

- ◯ Female
- ◯ Male

What is your date of birth?

Month ⌄ Year ⌄

What is your current marital status?

- ◯ Single or Never married
- ◯ Married
- ◯ Separated
- ◯ Divorced
- ◯ Widowed
- ◯ Prefer not to say

Are you currently pregnant?

- Yes
- No

Are you currently trying to get pregnant?

- Yes
- No
- I'm okay either way

Have you ever been pregnant and/or delivered a child?

- Yes
- No

Are you of Hispanic or Latino origin?

- Yes
- No

* Which category best describes your race?

- ◯ American Indian/Alaska Native
- ◯ Native Hawaiian/Pacific Islander
- ◯ Asian
- ◯ Black or African American
- ◯ White
- ◯ Other

* Were you born in the United States or U.S. territory?

- ◯ Yes
- ◯ No
- ◯ Prefer not to say

Where did you migrate from?

- West Africa
- North Africa
- Central Africa
- East Africa
- Southern Africa
- Caribbean/West Indies
- South America
- Central America
- North America (e.g., Canada)
- Europe
- Other (please specify)

What is your highest level of education completed?

- Completed grades 1-11
- 12th grade, no diploma
- High school or GED
- Some college
- Vocational or associate degree
- Bachelor's degree
- Master's degree
- Doctoral degree
- Other

What is your current employment status?

- ◯ Full-time employed
- ◯ Part-time employed
- ◯ Self-employed
- ◯ Not employed
- ◯ Retired
- ◯ Other

Are you currently a student enrolled in a postsecondary or vocational program?

- ◯ Yes, I am a full-time student
- ◯ Yes, I am a part-time student
- ◯ No, I am not a student

What was your household income before taxes last year?

- ◯ Less than $20,000
- ◯ $20,001 - $35,000
- ◯ $35,001 - $50,000
- ◯ $50,001 - $75,000
- ◯ $75,001 - $100,000
- ◯ Greater than $100,000
- ◯ I prefer not to say

Have you ever been diagnosed with the following health condition(s)? [check all that apply] If none, skip question.

- ☐ Asthma
- ☐ Type 1 diabetes
- ☐ Type 2 diabetes
- ☐ Gestational diabetes
- ☐ Hypertension (high blood pressure)
- ☐ Preeclampsia/eclampsia
- ☐ Cardiovascular disease
- ☐ Cancer
- ☐ Other chronic disease (please specify)

Are you currently covered by any kind of health insurance plan?

- ◯ Yes
- ◯ No
- ◯ Not sure

Which of these categories best describes how your health insurance plan was obtained?

- ◯ Private insurance bought through your job or the job of your partner or relative
- ◯ Private insurance bought from a health insurance plan (e.g., affordable care act)
- ◯ Tri-Care or other military coverage
- ◯ Medicaid/CHIP
- ◯ Other (please specify)

What is your height in feet and inches? (example, 5 feet 6 inches) *You will enter the number of __FEET__ here and the number of inches in the next field.*

- 3 feet
- 4 feet
- 5 feet
- 6 feet
- 7 feet
- 8 feet

What is your height in feet and inches? (example, 5 feet 6 inches) *You will enter the number of __INCHES__ here*

- 0 inches
- 1 inch
- 2 inches
- 3 inches
- 4 inches
- 5 inches
- 6 inches
- 7 inches
- 8 inches
- 9 inches
- 10 inches
- 11 inches

What is your current weight in pounds?

QuestionPro
